# Physics-Informed Simulation and Time-Series Classification of Ground-Based Infrared Radiant-Intensity Sequences for Space Objects

**DOI:** 10.3390/s26175335

**Published:** 2026-08-23

**Authors:** Yubo Wang, Shijun Song, Chun Jiang, Qiyang Gui, Tao Chen, Shuai Wang, Zhengwei Li

**Affiliations:** 1 Changchun Institute of Optics, Fine Mechanics and Physics, Chinese Academy of Sciences, Changchun 130033, China; 2 University of Chinese Academy of Sciences, Beijing 100049, China; 3 Jilin Provincial Key Laboratory of Intelligent Wavefront Sensing and Control, Changchun Institute of Optics, Fine Mechanics and Physics, Chinese Academy of Sciences, Changchun 130033, China

**Keywords:** infrared radiant-intensity sequence, space objects, physics-informed simulation, micromotion dynamics, time-series classification

## Abstract

Under ground-based observation geometry, infrared radiant-intensity sequences of space objects are jointly influenced by object micromotion, thermal radiation, time-varying viewing conditions, and atmospheric propagation. Existing simulation studies often prescribe the line of sight or simplify the coupling between viewing geometry and atmospheric attenuation, which limits long-duration ground-based sequence analysis. This study develops a physics-informed framework for generating atmosphere-attenuated infrared radiant-intensity sequences of space objects undergoing precession or tumbling. The framework reconstructs observation geometry from azimuth–elevation–range trajectories, updates facet normals through a unified micromotion attitude model, computes visible projected area and transient facet temperature, and incorporates MODTRAN-derived elevation-dependent atmospheric transmittance. Using this framework, we construct IRPeriodic, an eight-class simulated dataset for long-duration univariate time-series classification. We further propose LPD-Net, which integrates large-kernel residual feature extraction, prototype-guided dynamic temporal alignment, and differential periodic representation to capture long-range waveform morphology, sample-dependent temporal correspondence, and segment-level local variation. On IRPeriodic, LPD-Net achieves an accuracy of 0.8618 ± 0.0057, a macro-F1 of 0.8615 ± 0.0061, and a Matthews correlation coefficient of 0.8426 ± 0.0065, outperforming the evaluated neural-network and ROCKET-type baselines. Ablation and synthetic-noise sensitivity analyses indicate that the performance gain is mainly associated with long-context feature extraction, with additional improvements from dynamic alignment and differential periodic statistics. Auxiliary experiments on selected public UCR datasets suggest that the representation is also competitive for univariate time-series classification. These results demonstrate the effectiveness of LPD-Net on the proposed physics-informed benchmark for long-duration ground-based infrared radiant-intensity sequence classification.

## 1. Introduction

Infrared sensing provides temporal information for the analysis and classification of distant space objects. Object geometry, surface thermal state, attitude motion, and time-varying viewing conditions jointly modulate directional radiant intensity. In particular, precession, tumbling, and spin alter the visible projected area and thermal emission, producing characteristic temporal signatures [1,2]. High-sensitivity observation of weak and distant infrared objects is often supported by cooled infrared detectors and cold optical assemblies, which suppress detector dark current, instrument self-emission, and internal stray radiation [3,4,5]. The present study focuses on the object–trajectory–atmosphere coupling that determines the atmosphere-attenuated band-integrated directional radiant-intensity sequence, rather than on detector output or full sensor-chain modeling.

Physics-based infrared modeling has been widely used to connect temporal radiation signatures with object geometry, material properties, attitude motion, and thermal state [1,2,6,7,8]. Recent studies have also examined sequence-level infrared signatures and learning-based classification under long-range or noise-contaminated conditions [9]. Many controlled simulations, however, prescribe or simplify the line of sight so that micromotion-induced modulation can be isolated. This assumption is useful for controlled analysis, but it does not fully describe long-duration ground-based sequence generation, in which viewing direction and atmospheric path vary simultaneously.

Ground-based observation introduces a more coupled sequence-generation problem. Along each prescribed ground-based azimuth–elevation–range (AER) trajectory, the azimuth, elevation angle, range, line of sight (LOS), and atmospheric propagation path vary simultaneously. The elevation angle changes atmospheric transmittance, the viewing direction changes the projected area of each object facet, and the reconstructed object-position sequence determines the motion-direction reference frame used to describe micromotion. A fixed-viewing-direction model is therefore insufficient for generating ground-based infrared radiant-intensity sequences. A more suitable formulation should couple AER-driven observation geometry, micromotion attitude, projected-area variation, transient thermal radiation, and elevation-dependent atmospheric attenuation.

The present classification problem also differs from conventional infrared image classification. Existing infrared classification methods often rely on spatial texture, multi-scale image structure, spectral-band complementarity, channel attention, or ensemble representations [10,11,12,13,14]. These designs are effective when the input contains spatial, spectral, or multi-channel information. Related infrared moving-object classification studies have used radiant-intensity sequences and transfer learning to reduce limited-sample effects, but their settings mainly involve spatial infrared imagery and cross-domain transfer rather than AER-driven ground-based sequence generation with atmospheric attenuation [15]. In contrast, each sample in the present task is a normalized univariate radiant-intensity sequence. Its discriminative information is mainly expressed through long-duration waveform morphology, periodic fluctuation, phase-dependent temporal shifts, and local temporal variation.

General time-series classification methods provide important baselines for this problem [16,17,18]. Nevertheless, directly applying these methods may not capture all structures that matter in ground-based infrared radiant-intensity sequences. Global pooling can suppress segment-level differences, fixed segmentation can place similar micromotion stages at different temporal positions across samples, and short-context feature extraction can miss long-range waveform morphology. A classification model for this task should therefore capture long temporal context, establish sample-dependent temporal correspondence, and preserve local variation within dynamically matched temporal stages.

To address these modeling and classification requirements, this study develops a physics-informed framework for simulation and time-series classification of ground-based infrared radiant-intensity sequences for space objects. The physical model reconstructs viewing geometry from AER trajectories, defines object-centered coordinate frames from the reconstructed position sequence, describes precession and tumbling under a unified attitude convention, computes facet-level projected area and transient thermal radiation, and applies MODTRAN-derived elevation-dependent atmospheric attenuation. The generated sequences preserve the coupling among object geometry, micromotion attitude, observation geometry, thermal radiation, and atmospheric path variation.

Based on this framework, an eight-class simulated dataset, IRPeriodic, is constructed for time-series classification. The dataset includes flat-base cone, cone–cylinder composite, spherical-base cone, cylindrical, and hexagonal-prism geometries under precession or tumbling. Each sample is an atmosphere-attenuated 80 s univariate infrared radiant-intensity sequence sampled at 25 Hz, giving 2000 temporal points. In total, IRPeriodic contains 640 samples per class and 5120 samples generated from geometric parameters, micromotion settings, and 80 prescribed ground-based AER trajectories. The dataset is designed to evaluate whether sequence classifiers can separate physically related object classes when waveform morphology, phase shift, local fluctuation, and atmospheric attenuation are jointly involved.

For classification, this study proposes LPD-Net, a one-dimensional network tailored to long-duration infrared radiant-intensity sequences. LPD-Net uses large-kernel residual convolution blocks to extract long-context temporal features from 2000 to point sequences. It then introduces prototype-guided dynamic temporal alignment to establish sample-dependent correspondence between latent temporal stages and deep feature positions. A differential periodic representation finally summarizes each dynamically aligned prototype-level representation through weighted fluctuation magnitude and adjacent-time variation. This design follows the structure of the signal: class information extends over broad temporal intervals, but it is expressed through local variations whose positions may shift across samples.

Experiments on IRPeriodic show that LPD-Net achieves the best overall performance among the compared recurrent, convolutional, InceptionTime, and ROCKET-type baselines. It obtains an accuracy of 0.8618 ± 0.0057, a macro-F1 of 0.8615 ± 0.0061, and a Matthews correlation coefficient of 0.8426 ± 0.0065. Ablation studies show that the large-kernel residual backbone provides the main performance gain, while prototype-guided alignment and differential periodic statistics further improve temporal correspondence and local-variation representation. Synthetic additive-noise sensitivity tests and auxiliary public UCR evaluations are also conducted to analyze sensitivity, auxiliary behavior, and limitations of the representation.

The main contributions of this study are summarized as follows:

1. A physics-informed ground-based infrared radiant-intensity sequence-generation framework is established by coupling AER-driven observation geometry, micromotion attitude, projected-area calculation, transient thermal radiation, and MODTRAN-derived atmospheric attenuation.
2. An eight-class simulated dataset, IRPeriodic, is constructed for simulated space-object time-series classification, with each sample represented as an atmosphere-attenuated 80 s univariate infrared radiant-intensity sequence.
3. LPD-Net is proposed for long-duration infrared sequence classification by integrating large-kernel residual feature extraction, prototype-guided dynamic temporal alignment, and differential periodic representation.
4. A set of experiments, including baseline comparison, class-level analysis, synthetic additive-noise sensitivity tests, ablation studies, kernel and temporal-prototype-number analyses, and auxiliary UCR evaluations, is conducted to assess the effectiveness and limitations of the temporal representation.

## 2. Physics-Informed Ground-Based Infrared Sequence Generation

**Figure 1:**
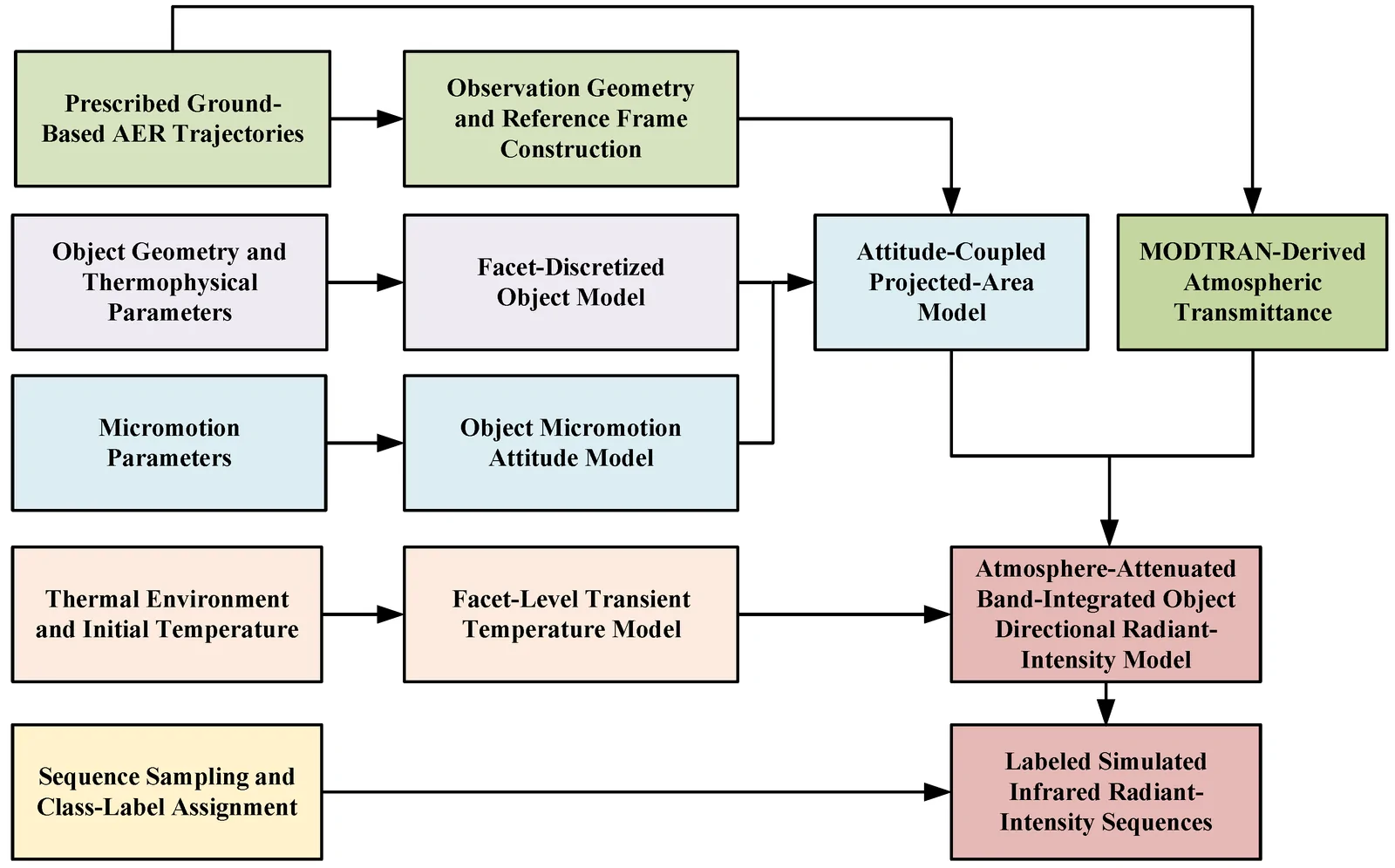
Physics-informed workflow for generating labeled simulated infrared radiant-intensity sequences of space objects under ground-based observation.

Infrared radiant-intensity sequences of space objects are governed by object geometry, micromotion attitude, projected area, surface temperature, and observation geometry [1,2]. In ground-based observation, these factors are coupled with a time-varying atmospheric path. The sequence-generation model used to construct IRPeriodic therefore reconstructs the viewing geometry from azimuth–elevation–range (AER) trajectories, updates facet normals according to object micromotion, computes visible projected area and thermal radiation, and applies elevation-dependent atmospheric attenuation. The overall simulation workflow is summarized in Figure 1.

### 2.1. Observation Geometry and Coordinate Frames

**Figure 2:**
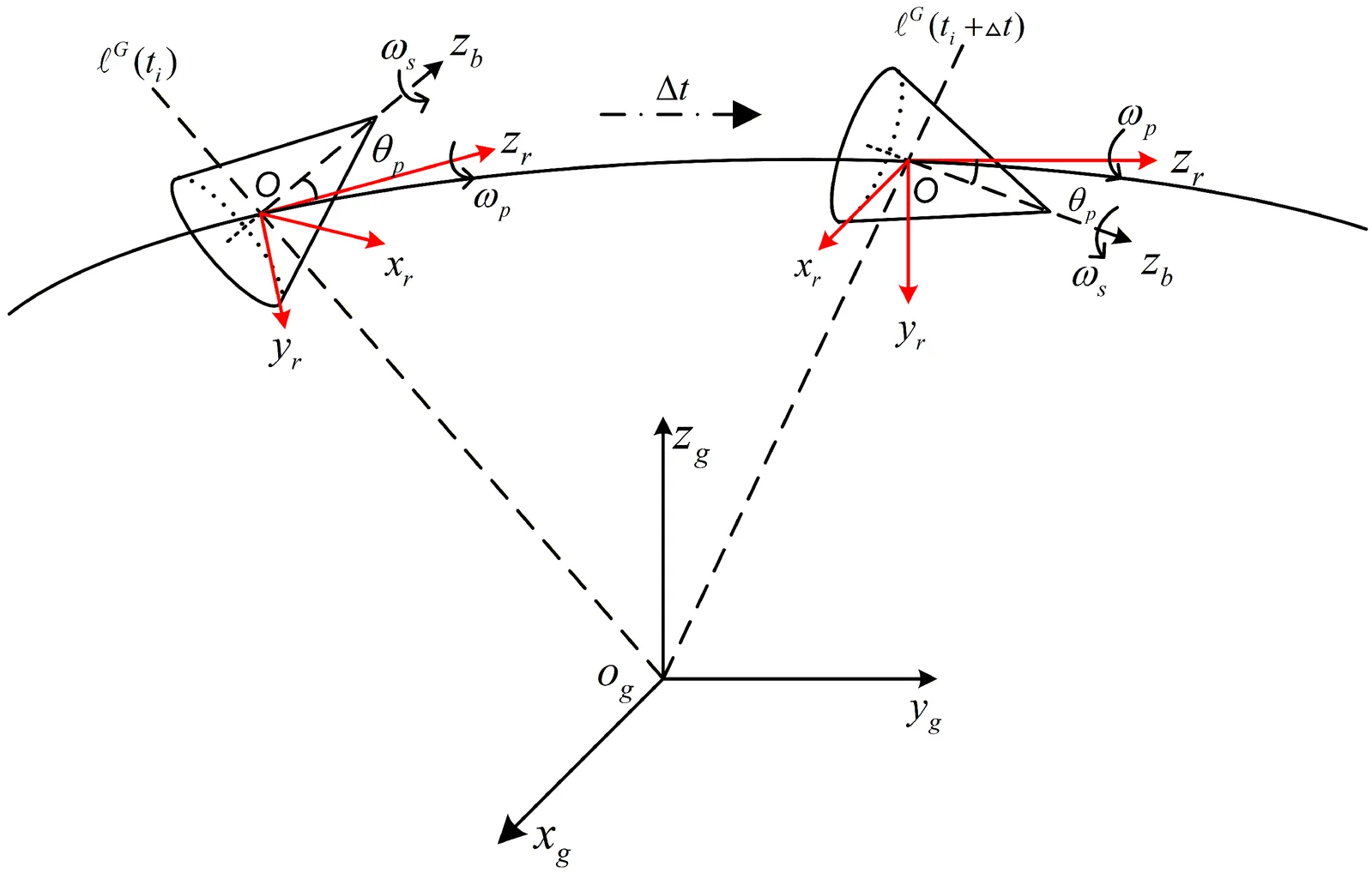
Coordinate frames and LOS geometry for ground-based observation of a space object.

Three frames are used: the object body-fixed frame *B*:$o_{b}-x_{b}y_{b}z_{b}$; the object-centered reference frame $R:o_{r}-x_{r}y_{r}z_{r}$; and the station-centered observation frame *G*:$o_{g}-x_{g}y_{g}z_{g}$. The origin $o_{b}$ is located at the object centroid, and the $z_{b}$-axis is aligned with the principal axis of the object. The origin $o_{g}$ is located at the observation station; the $x_{g}$- and $y_{g}$-axes span the local horizontal plane, and the $z_{g}$-axis points to the local zenith. The coordinate-frame convention and the time-varying line-of-sight vector $\ell^{G}\left(t\right)$ used in the micromotion model are illustrated in Figure 2.

For the coordinate transformations used in this study, a coordinate-frame superscript denotes the frame in which a directional vector is expressed. The rotation matrix follows the destination–source convention, namely,

(1) $$v^{A}=R_{AB}v^{B},$$

where $R_{AB}$ maps vector coordinates from frame *B* to frame *A*. Let $\phi_{o}\left(t\right)$, $\theta_{o}\left(t\right)$, and $R_{o}\left(t\right)$ denote the ground-based observation azimuth, elevation angle, and range, respectively. The observation azimuth $\phi_{o}\left(t\right)$ is measured in the ground $x_{g}$–$y_{g}$ plane from $+x_{g}$ toward $+y_{g}$, and the elevation $\theta_{o}\left(t\right)$ is measured from the local horizontal plane. Under this convention, the LOS unit vector from the observation station to the object centroid and the corresponding viewing-direction vector from the object centroid to the station are

(2) $$\ell^{G}\left(t\right)=\left[\begin{array}{c} \cos\theta_{o}\left(t\right)\cos\phi_{o}\left(t\right)\\ \cos\theta_{o}\left(t\right)\sin\phi_{o}\left(t\right)\\ \sin\theta_{o}\left(t\right) \end{array}\right],\;s^{G}\left(t\right)=-\ell^{G}\left(t\right).$$

The object-centroid position in frame *G* is reconstructed as

(3) $$p_{o}^{G}\left(t\right)=R_{o}\left(t\right)\ell^{G}\left(t\right).$$

For a discrete trajectory ${\left\{p_{o}^{G}\left(t_{i}\right)\right\}}_{i=1}^{N}$, the instantaneous motion direction is estimated by a central finite difference for interior samples and by one-sided differences at the two endpoints:

(4) $$v_{o}^{G}\left(t_{i}\right)=\left\{\begin{array}{cc} \frac{p_{o}^{G}\left(t_{i+1}\right)-p_{o}^{G}\left(t_{i}\right)}{t_{i+1}-t_{i}}, & i=1,\\ \frac{p_{o}^{G}\left(t_{i+1}\right)-p_{o}^{G}\left(t_{i-1}\right)}{t_{i+1}-t_{i-1}}, & 1<i<N,\\ \frac{p_{o}^{G}\left(t_{i}\right)-p_{o}^{G}\left(t_{i-1}\right)}{t_{i}-t_{i-1}}, & i=N, \end{array}\right.\;e_{v}^{G}\left(t_{i}\right)=\frac{v_{o}^{G}\left(t_{i}\right)}{∥v_{o}^{G}\left(t_{i}\right)∥}.$$

In this formulation, the $z_{r}$-axis of frame *R* is aligned with $e_{v}^{G}\left(t\right)$. A non-collinear auxiliary vector $b^{G}$ is selected from the local-horizon frame, and the axes of frame *R* expressed in frame *G* are constructed as

(5) $$e_{z_{r}}^{G}\left(t\right)=e_{v}^{G}\left(t\right),\;e_{x_{r}}^{G}\left(t\right)=\frac{b^{G}\times e_{z_{r}}^{G}\left(t\right)}{∥b^{G}\times e_{z_{r}}^{G}\left(t\right)∥},\;e_{y_{r}}^{G}\left(t\right)=e_{z_{r}}^{G}\left(t\right)\times e_{x_{r}}^{G}\left(t\right).$$

Thus, the rotation matrix from frame *R* to frame *G* is

(6) $$R_{GR}\left(t\right)=\left[\begin{array}{ccc} e_{x_{r}}^{G}\left(t\right) & e_{y_{r}}^{G}\left(t\right) & e_{z_{r}}^{G}\left(t\right) \end{array}\right].$$

### 2.2. Micromotion and Projected-Area Model

The object surface is discretized into $N_{s}$ facets. For the *j*-th facet, the area, outward unit normal vector in frame *B*, and material-parameter vector are denoted by $A_{j}$, $n_{j}^{B}$, and $m_{j}$, respectively. The vector $m_{j}$ contains the optical and thermophysical parameters used in the heat-balance and radiation models. Cone, cylinder, spherical-base, end-surface, and prism components are all represented through the unified facet set

(7) $$S_{k}={\left\{\left(A_{j},n_{j}^{B},m_{j}\right)\right\}}_{j=1}^{N_{s}},\;k=1,2,\ldots ,K.$$

This notation keeps the geometric discretization independent of a particular object shape, while retaining the facet areas and normals required for projected-area and thermal-radiation calculations.

Micromotion is described by a compact Rodrigues rotation operator. For a unit axis $a^{F}$ expressed in frame *F* and a rotation angle ψ,

(8) $$R\left(a^{F},\psi \right)=I_{3}\cos\psi +\left(1-\cos\psi \right)a^{F}{\left(a^{F}\right)}^{T}+{\left[a^{F}\right]}_{\times }\sin\psi ,$$

where ${\left[a^{F}\right]}_{\times }$ is the skew-symmetric matrix satisfying ${\left[a^{F}\right]}_{\times }b=a^{F}\times b$.

For precession, the $z_{r}$-axis is taken as the coning axis and is aligned with the instantaneous motion direction. The body symmetry axis $z_{b}$ forms the precession angle $\theta_{p}$ with $z_{r}$, and $u_{p,0}^{R}$ denotes its initial direction in frame *R*. With coning angular velocity $\omega_{p}$ and spin angular velocity $\omega_{s}$, the precession attitude is modeled by a spin rotation about the initially tilted body axis and a coning rotation about $e_{z_{r}}^{R}={\left[0,0,1\right]}^{T}$. Under the column-vector convention adopted in this work, the rightmost rotation acts first. Therefore, the spin rotation is first applied, and the resulting attitude is then coned about the $z_{r}$-axis. This product keeps the body symmetry axis on the prescribed precession cone while allowing spin about the body axis. For tumbling, $\theta_{t}$ specifies the inclination of the fixed tumbling axis $u_{t}^{R}$ in frame *R* rather than a time-varying tumbling angle. Once $u_{t}^{R}$ is determined by $\theta_{t}$ and the initial phase, the object rotates about this fixed axis with angular velocity $\omega_{t}$. The resulting micromotion rotation matrix is

(9) $$R_{m}^{R}\left(t\right)=\left\{\begin{array}{cc} R\left(e_{z_{r}}^{R},\omega_{p}t\right)R\left(u_{p,0}^{R},\omega_{s}t\right), & \mathrm{precession},\\ R(u_{t}^{R},\omega_{t}t), & \mathrm{tumbling}. \end{array}\right.$$

The initial azimuthal phase of the micromotion model is fixed at $\varphi_{0}=7\pi /4$ for all eight object classes. For coning objects, the initial tilted body-axis direction is constructed using $\varphi_{0}$; equivalently, the coning phase evolves as $\varphi_{0}+\omega_{p}t$, while the spin rotation is governed by $\omega_{s}t$. For tumbling objects, the tumbling axis $u_{t}^{R}$ is constructed from the first-frame object-motion azimuth and elevation, and the initial body orientation uses the same $\varphi_{0}$. This convention fixes the phase reference used in the sequence generation and avoids introducing an additional phase symbol that could be confused with the channel-wise gate α used later in LPD-Net. The time-varying attitude matrix and the *j*-th facet normal expressed in the ground-based frame are

(10) $$R_{RB}\left(t\right)=R_{m}^{R}\left(t\right)R_{RB,0},\;n_{j}^{G}\left(t\right)=R_{GR}\left(t\right)R_{RB}\left(t\right)n_{j}^{B}.$$

Here, the essential coupling is that the AER trajectory determines the dynamic LOS and the object-centered frame, while micromotion updates the facet normals; together, they determine the visible projected area. For the closed convex target geometries used in IRPeriodic, the visible projected area of the *j*-th facet along the viewing direction is

(11) $$A_{\mathrm{proj},j}\left(t\right)=A_{j}\max\left[0,n_{j}^{G}\left(t\right)\cdot s^{G}\left(t\right)\right].$$

This projected-area formulation assumes that a facet contributes when its outward normal faces the viewing direction and contributes zero otherwise. It is suitable for the simplified cone, cone–cylinder, spherical-base cone, cylinder, and prism geometries considered in this dataset, but more complex non-convex structures would require explicit visibility or ray-tracing treatment. The total visible projected area is

(12) $$A_{\mathrm{proj}}\left(t\right)=\sum_{j=1}^{N_{s}}A_{\mathrm{proj},j}\left(t\right).$$

### 2.3. Thermal Radiation and Atmospheric Attenuation

The object temperature field is represented by the transient facet temperature sequence ${\left\{T_{j}\left(t\right)\right\}}_{j=1}^{N_{s}}$. The initial temperature is prescribed by the simulation setting, denoted as

(13) $$T_{j}\left(0\right)=T_{j,0},\;j=1,2,\ldots ,N_{s}.$$

For all object classes and facets, a uniform initial temperature of $T_{j,0}=300\;K$ is used. For the *j*-th facet, the heat-balance equation is

(14) $$C_{j}\frac{dT_{j}\left(t\right)}{dt}=Q_{\mathrm{sun},j}\left(t\right)+Q_{\mathrm{earth},j}\left(t\right)+Q_{\mathrm{ref},j}\left(t\right)+Q_{\mathrm{cond},j}\left(t\right)-Q_{\mathrm{rad},j}\left(t\right),$$

where $C_{j}$ is the equivalent heat capacity of the facet. The terms on the right-hand side represent absorbed solar radiation, Earth infrared radiation, Earth-reflected solar radiation, conductive exchange, and radiative loss to the cold-space background, respectively. These are standard thermal-radiation components; in this work, they are used to obtain the transient facet temperature field that drives the infrared radiant-intensity sequence. For a thin-wall facet, the principal thermal terms used in the present model can be written as

(15) $$\begin{array}{cccc} C_{j} & =\rho_{j}c_{j}\delta_{j}A_{j}, & Q_{\mathrm{rad},j}\left(t\right) & =\varepsilon_{j}\sigma A_{j}T_{j}^{4}\left(t\right),\\ Q_{\mathrm{sun},j}\left(t\right) & =\alpha_{s,j}E_{s}A_{j}F_{\mathrm{sun},j}\left(t\right), & Q_{\mathrm{earth},j}\left(t\right) & =\alpha_{e,j}E_{e}A_{j}F_{\mathrm{earth},j}\left(t\right),\\ Q_{\mathrm{ref},j}\left(t\right) & =\alpha_{s,j}\rho_{e}E_{s}A_{j}F_{\mathrm{ref},j}\left(t\right). & \; & \; \end{array}$$

Here, $\rho_{j}$, $c_{j}$, and $\delta_{j}$ are the density, specific heat, and equivalent thickness, respectively; $\alpha_{s,j}$ is the solar-band absorptivity; $E_{s}$ and $E_{e}$ are the solar and Earth-infrared irradiances, respectively; $\rho_{e}$ is the mean Earth albedo; and $F_{\mathrm{sun},j}$, $F_{\mathrm{earth},j}$, and $F_{\mathrm{ref},j}$ are time-dependent irradiation factors determined by the facet orientation and Sun–Earth geometry. For the Earth-infrared heating term, the long-wave absorptivity is approximated by the corresponding infrared emissivity, i.e., $\alpha_{e,j}\approx \varepsilon_{j}$, under the adopted long-wave gray-surface approximation. The representative solar absorptivities and infrared emissivities used in the simulation are summarized in Table 1 and selected with reference to the cited material-property literature [19,20].

A graybody assumption is used in the 8–12 μm band, so the facet emissivity is treated as wavelength independent within this band. According to Planck’s law,

(16) $$L_{\lambda }\left(T\right)=\frac{2h_{P}c_{0}^{2}}{\lambda^{5}\left[\exp\left(\frac{h_{P}c_{0}}{k_{B}T\lambda }\right)-1\right]},$$

where $h_{P}$, $c_{0}$, and $k_{B}$ are Planck’s constant, the speed of light in vacuum, and Boltzmann’s constant, respectively. The band-integrated radiance of the *j*-th facet is

(17) $$L_{8-12,j}\left(t\right)=\int_{\lambda_{1}}^{\lambda_{2}}\varepsilon_{j}L_{\lambda }\left[T_{j}\left(t\right)\right]d\lambda ,\;\lambda_{1}=8\;\mu m,\;\lambda_{2}=12\;\mu m.$$

The object directional radiant intensity before atmospheric attenuation is obtained by summing the band-integrated contribution of all visible facets:

(18) $$I_{\mathrm{obj}}\left(t\right)=\sum_{j=1}^{N_{s}}L_{8-12,j}\left(t\right)A_{\mathrm{proj},j}\left(t\right).$$

Atmospheric attenuation is a key factor in ground-based infrared observation because the propagation path varies with elevation angle. MODTRAN v5.2.1 is adopted here to calculate elevation-dependent band-averaged transmittance under the baseline atmospheric settings used in IRPeriodic [21,22]. In the present sequence-generation framework, MODTRAN is used to calculate the atmospheric transmittance applied to the target directional radiant-intensity component. Accordingly, I(t) represents the transmitted target component, while atmospheric path emission pertains to a more complete at-sensor radiometric formulation. The elevation-dependent atmospheric transmittance is implemented as a band-averaged engineering approximation in the present sequence-generation model, and the same approximation is applied consistently to all generated samples. The elevation–transmittance lookup table is

(19) $$D_{\tau }={\left\{\left(\theta_{q},{\bar{\tau }}_{q}\right)\right\}}_{q=1}^{N_{\tau }},\;{\bar{\tau }}_{q}=\frac{1}{\lambda_{2}-\lambda_{1}}\int_{\lambda_{1}}^{\lambda_{2}}\tau_{\lambda }\left(\theta_{q}\right)d\lambda .$$

The transmittance at time *t* is obtained by interpolation over the observation elevation angle,

(20) $$\tau \left(t\right)=I_{\theta }\left(D_{\tau };\theta_{o}\left(t\right)\right),\;I\left(t\right)=\tau \left(t\right)I_{\mathrm{obj}}\left(t\right).$$

The model output I(t) is defined as the atmosphere-attenuated band-integrated object directional radiant intensity, with units of W/sr. Sensor-aperture irradiance, geometric dilution by $1/R_{o}^{2}\left(t\right)$, and detector response are not included in this signal definition. The range $R_{o}\left(t\right)$ is used to reconstruct the object position, motion-direction reference frame, and viewing geometry.

### 2.4. IRPeriodic Dataset Construction

The dataset is generated by sampling object class, micromotion parameters, and ground-based observation trajectory. For each parameter combination, the preceding equations produce a complete atmosphere-attenuated directional radiant-intensity sequence. The sequence-level dataset settings, including the spectral band, atmospheric configuration, sequence length, and dataset scale, are summarized in Table 2. The dataset contains eight geometry–micromotion classes, and their geometric, micromotion, and thermophysical parameters are summarized in Table 1.

The observer altitude of 3.2 km was selected with reference to the Gaomeigu site of Lijiang Observatory, Yunnan Observatories, Chinese Academy of Sciences, at an altitude of 3193 m [23]. The altitude was rounded to 3.2 km for the MODTRAN atmospheric-transmission calculation. The prescribed AER trajectories remain independently specified.

The AER trajectory set contains 80 prescribed ground-based AER trajectories representing different viewing conditions, and it is fixed before sequence generation. Each labeled sample is then defined as a fixed-length time window of the atmosphere-attenuated directional radiant-intensity sequence. The *m*-th sample is expressed as

(21) $$\begin{array}{cc} x_{m} & ={\left[I\left(t_{m,1}\right),I\left(t_{m,2}\right),\ldots ,I\left(t_{m,L}\right)\right]}^{T},\\ y_{m} & \in \{1,2,\ldots ,K\},\;L=2000,\;K=8. \end{array}$$

**Figure 3:**
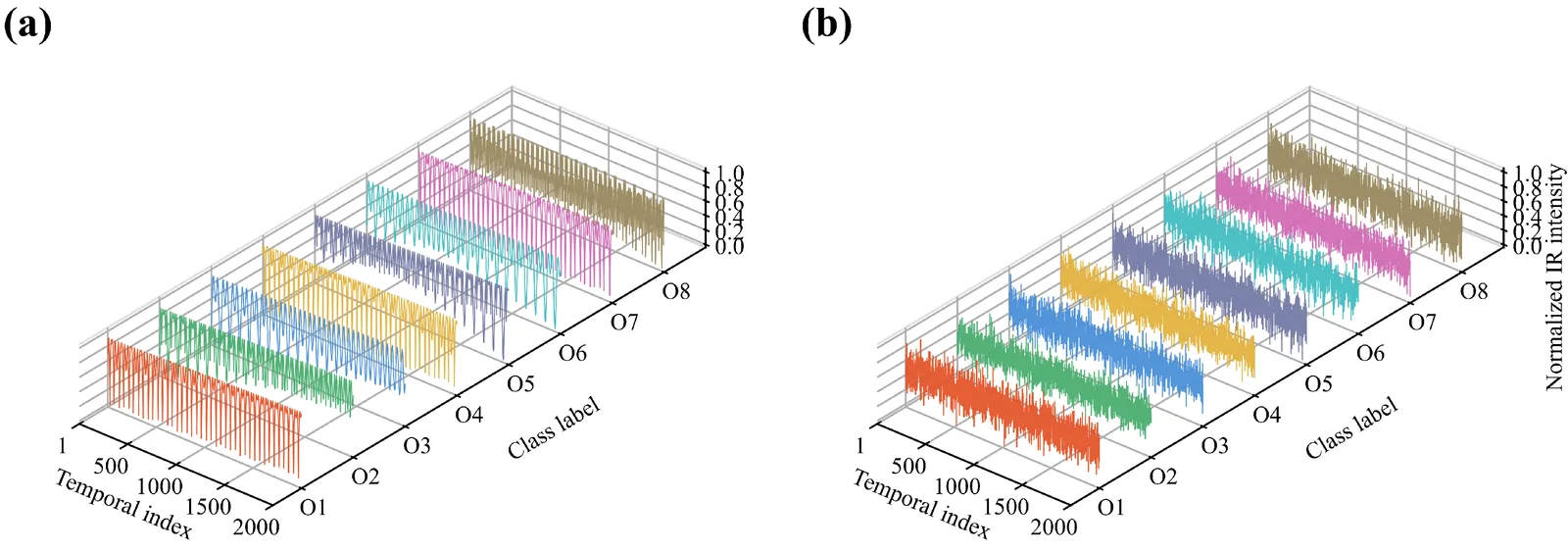
Normalized infrared radiant-intensity sequences of eight object classes: (**a**) noise-free sequences; (**b**) sequences contaminated by additive white Gaussian noise (AWGN) at a SNR of 20 dB.

Each value in Equation (21) is generated from the physical chain of AER geometry, micromotion attitude, temperature field, projected area, band-integrated radiation, and atmospheric attenuation. The resulting labeled time-series samples are used in subsequent classification experiments; the normalized infrared radiant-intensity sequences of the eight object classes under noise-free and noise-contaminated conditions are shown in Figure 3.

## 3. LPD-Net for Infrared Radiant-Intensity Sequence Classification

**Figure 4:**
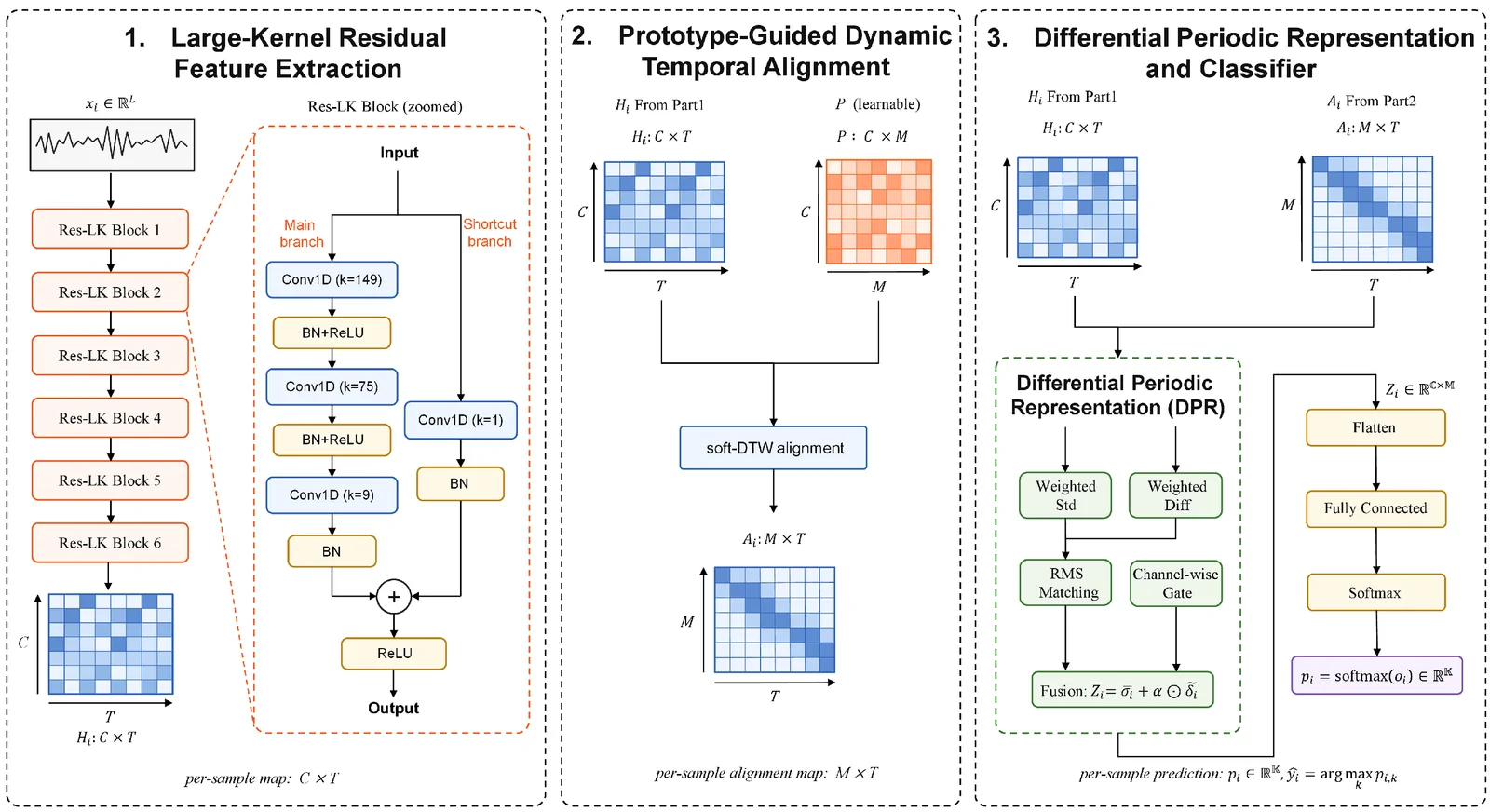
Overall architecture of LPD-Net for infrared radiant-intensity sequence classification.

The generated infrared radiant-intensity sequences are long-duration univariate time series. Their class information appears in global waveform morphology, phase-dependent modulation, and local temporal variation. LPD-Net models these three aspects with a large-kernel residual backbone, prototype-guided dynamic temporal alignment, and a differential periodic representation. The overall network architecture is shown in Figure 4.

### 3.1. Input Representation

An infrared radiant-intensity sequence is represented as a one-dimensional real-valued signal ordered by time. The *i*-th sample is denoted as

(22) $$x_{i}=\left[x_{i,1},x_{i,2},\ldots ,x_{i,L}\right]\in R^{L},$$

where $x_{i,t}$ is the infrared radiant-intensity value at the *t*-th sampling instant and *L* is the sequence length. The sample label is denoted as

(23) $$y_{i}\in \left\{1,2,\ldots ,K\right\},$$

where *K* is the number of object classes. For mini-batch input, the sequences are arranged as the tensor

(24) $$X\in R^{B\times 1\times L},$$

where *B* is the batch size and the channel number is 1. This input form preserves the original temporal index and allows one-dimensional convolution kernels to aggregate local and long-range temporal features along the time axis.

The absolute radiant-intensity scale may vary among samples. The input sequence is normalized before entering the network, and the normalized sample is still denoted by $x_{i}$. Normalization changes only the amplitude scale and does not change temporal order or class labels. Therefore, the discriminative information learned by the network still comes from sequence morphology, segment fluctuation, and adjacent-time variation.

### 3.2. Feature Extraction

LPD-Net maps the input tensor to a segment-level temporal statistical representation. LPD-Net separates feature extraction, temporal correspondence, and segment-level statistics so that each operation targets a different property of the sequence. The overall computation is

(25) $$H=F_{L}\left(X\right),$$

(26) $$A=F_{P}\left(H;P\right),$$

(27) $$Z=F_{D}\left(H,A\right),$$

where $F_{L}\left(\cdot \right)$ denotes the large-kernel residual feature extraction function, and $H\in R^{B\times C\times T}$ is the deep temporal feature map. $F_{P}\left(\cdot \right)$ denotes the prototype-guided dynamic temporal alignment function, $P\in R^{C\times M}$ is the learnable temporal prototype matrix, and $A\in R^{B\times M\times T}$ is the alignment matrix. $F_{D}\left(\cdot \right)$ denotes the differential periodic representation function, and $Z\in R^{B\times C\times M}$ is the segment-level representation before classification. Here, *C* is the number of deep feature channels, *T* is the temporal length of the deep feature map, and *M* is the number of learnable temporal prototypes. It should not be interpreted as a fixed equal-length partition of the input sequence.

#### 3.2.1. Large-Kernel Residual Convolution Block

The large-kernel residual convolution block maps the raw sequence to deep temporal features. Discriminative information in infrared radiant-intensity sequences may span a long temporal interval; short convolution kernels require deeper stacking to cover the same range. LPD-Net uses large one-dimensional convolution kernels to aggregate broad temporal context within each residual block, while residual connections improve the optimization stability of the deep network.

Let $H^{\left(b-1\right)}$ be the input of the *b*-th residual block. The block consists of three one-dimensional convolutional layers and one shortcut branch. The convolutional branch is computed as

(28) $$U_{1}^{\left(b\right)}=\phi \left(\mathrm{BN}\left({\mathrm{Conv}}_{k_{1}}\left(\mathrm{Pad}\left(H^{\left(b-1\right)}\right)\right)\right)\right),$$

(29) $$U_{2}^{\left(b\right)}=\phi \left(\mathrm{BN}\left({\mathrm{Conv}}_{k_{2}}\left(\mathrm{Pad}\left(U_{1}^{\left(b\right)}\right)\right)\right)\right),$$

(30) $$U_{3}^{\left(b\right)}=\mathrm{BN}\left({\mathrm{Conv}}_{k_{3}}\left(\mathrm{Pad}\left(U_{2}^{\left(b\right)}\right)\right)\right),$$

where ${\mathrm{Conv}}_{k}$ is a one-dimensional convolution with kernel length *k*, BN(·) is batch normalization, ϕ(·) is the ReLU activation function, and Pad(·) is zero padding that preserves temporal length. The shortcut branch uses a 1×1 convolution for channel matching:

(31) $$S^{\left(b\right)}=\mathrm{BN}\left({\mathrm{Conv}}_{1}\left(H^{\left(b-1\right)}\right)\right).$$

The output of the residual block is

(32) $$H^{\left(b\right)}=\phi \left(U_{3}^{\left(b\right)}+S^{\left(b\right)}\right).$$

The LPD-Net backbone is composed of six stacked large-kernel residual blocks. The structural parameters of each residual block and the backbone channel configuration are summarized in Table 3 and Table 4, respectively. Each residual block uses the kernel combination (149,75,9), with stride 1 in all convolutional layers. Under this setting, the theoretical receptive-field increment of one residual block is (149−1)+(75−1)+(9−1), and the theoretical receptive field of six residual blocks is R=1+6[(149−1)+(75−1)+(9−1)]=1381. Ignoring boundary-padding effects, this receptive field describes the input temporal range received by one deep feature position and provides long temporal context for dynamic alignment and segment-level statistics.

#### 3.2.2. Prototype-Guided Dynamic Temporal Alignment

**Figure 5:**
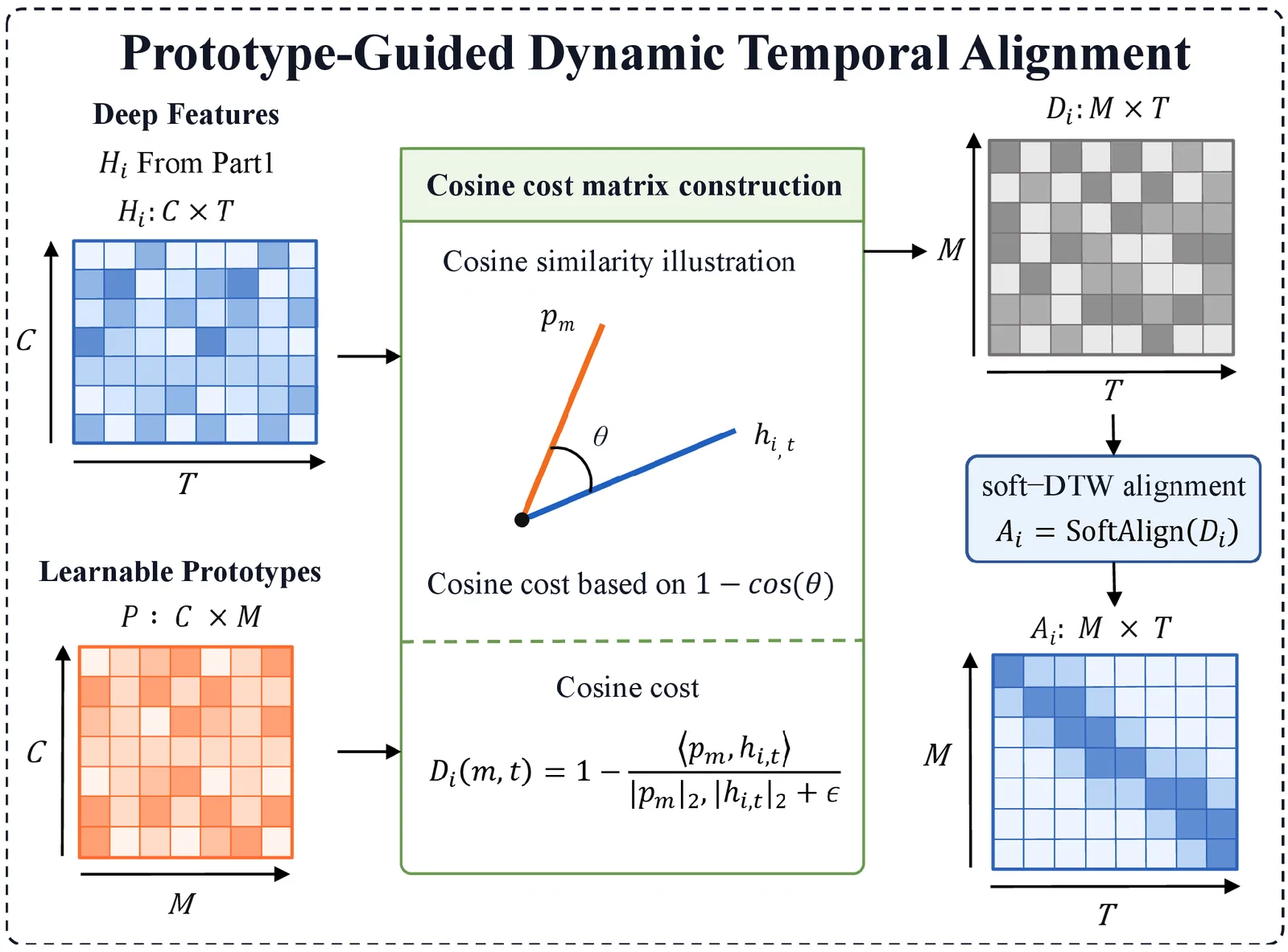
Prototype-guided dynamic temporal alignment. The module constructs a cosine prototype–feature cost matrix and performs soft alignment based on soft dynamic time warping (Soft-DTW) to obtain $A_{i}$, a soft correspondence matrix between *M* learnable temporal prototypes and *T* feature positions.

The feature map H produced by the large-kernel residual backbone retains the temporal dimension. Direct global pooling compresses structural differences among temporal stages into a single vector, whereas fixed equal-length segmentation may assign semantically similar stages in different samples to different intervals. LPD-Net introduces prototype-guided dynamic temporal alignment to form sample-dependent soft temporal correspondence in the feature space. The detailed structure of this alignment module is shown in Figure 5.

Let the backbone output be

(33) $$H\in R^{B\times C\times T}.$$

LPD-Net defines learnable temporal prototypes as

(34) $$P=\left[p_{1},p_{2},\ldots ,p_{M}\right]\in R^{C\times M},$$

where $p_{m}\in R^{C}$ denotes the *m*-th temporal prototype. The prototype index represents learnable temporal prototypes rather than class centers or fixed equal-length intervals. Therefore, the dimension of P is C×M, not K×C×M.

For the *i*-th sample, let $h_{i,t}\in R^{C}$ denote the deep feature at the *t*-th temporal position. The local matching cost between a temporal prototype and the feature sequence is

(35) $$D_{i,m,t}=d\left(p_{m},h_{i,t}\right),$$

where d(·,·) is a local distance function. Cosine distance is used to construct the local cost matrix $D_{i}$:

(36) $$d\left(p_{m},h_{i,t}\right)=1-\frac{p_{m}^{⊤}h_{i,t}}{{∥p_{m}∥}_{2}{∥h_{i,t}∥}_{2}+\epsilon }.$$

Collecting all pairwise costs gives the local cost matrix $D_{i}=\left[D_{i,m,t}\right]\in R^{M\times T}$. Here, $D_{i}$ denotes the local prototype–feature cost matrix used for Soft-DTW alignment and should not be confused with the differential periodic representation module. Dynamic time warping (DTW) provides a classical dynamic-programming framework for temporal alignment [24]. Soft-DTW replaces the hard minimum path selection in conventional DTW with a smooth minimum operator, making the alignment objective differentiable [25]:

(37) $${\mathrm{softmin}}_{\gamma }\left(a_{1},\ldots ,a_{n}\right)=-\gamma \log\sum_{j=1}^{n}\exp\left(-a_{j}/\gamma \right),$$

where γ controls the smoothness of the approximation and was set to 1.0 in all experiments.

Based on the Soft-DTW alignment result, the alignment matrix of the *i*-th sample is

(38) $$A_{i}=\mathrm{SoftAlign}\left(D_{i}\right),\;A_{i}\in R^{M\times T}.$$

Based on the prototype–feature cost matrix $D_{i}$, SoftAlign(·) denotes the Soft-DTW-based soft-alignment operation that produces a soft correspondence matrix $A_{i}$. The matrix $A_{i}$ is the soft alignment, or backward alignment, weight matrix returned by Soft-DTW, rather than the scalar Soft-DTW distance. The element $A_{i,m,t}$ denotes the contribution weight of the *t*-th feature position to the *m*-th temporal prototype. Thus, M=4 in this study represents four learnable temporal prototypes rather than four fixed equal-length temporal segments. The alignment matrix establishes soft temporal correspondence through prototype–feature matching and provides sample-dependent dynamic segment weights for the subsequent differential periodic representation.

#### 3.2.3. Differential Periodic Representation Module

**Figure 6:**
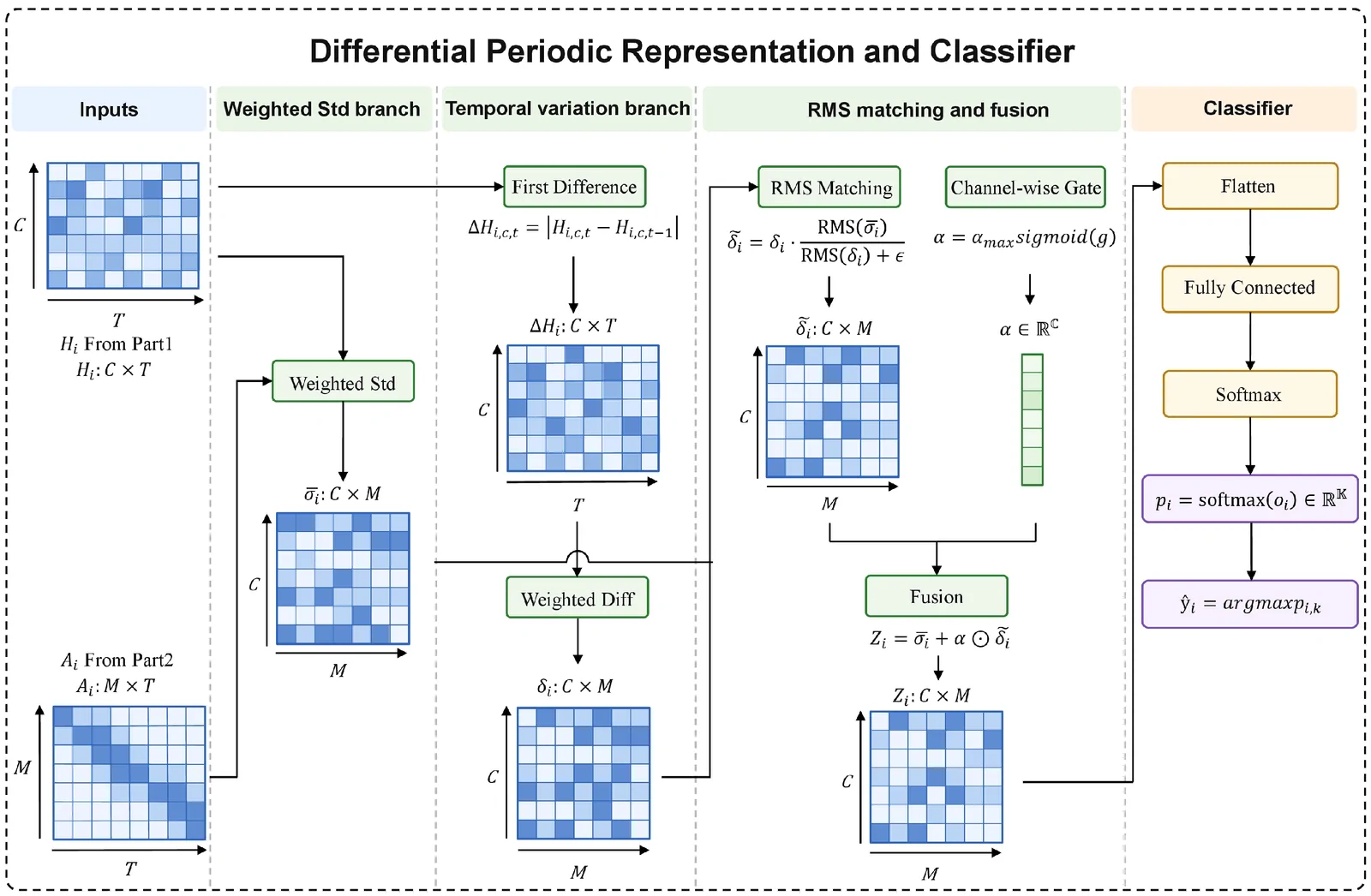
Detailed architecture of the differential periodic representation module and classifier.

The differential periodic representation module converts the dynamic alignment result into a fixed-dimensional representation. A single fluctuation statistic cannot distinguish whether a segment varies smoothly or through rapid adjacent-time changes, so the module combines weighted standard deviation with weighted first absolute difference. For each temporal segment, it computes two statistics: weighted standard deviation, which describes feature fluctuation magnitude within the segment, and weighted first absolute difference, which describes local variation strength between adjacent temporal positions. The detailed structure of this module is illustrated in Figure 6.

For sample *i* and segment *m*, the segment-weight normalization factor is

(39) $$q_{i,m}=\sum_{t=1}^{T}A_{i,m,t}+\epsilon ,$$

where ϵ is a constant used to avoid division by zero. The weighted mean within the segment is

(40) $$\mu_{i,c,m}=\frac{1}{q_{i,m}}\sum_{t=1}^{T}A_{i,m,t}H_{i,c,t}.$$

The weighted standard deviation within the segment is

(41) $$\sigma_{i,c,m}=\sqrt{\frac{1}{q_{i,m}}\sum_{t=1}^{T}A_{i,m,t}{\left(H_{i,c,t}-\mu_{i,c,m}\right)}^{2}+\epsilon }.$$

The weighted standard-deviation map is denoted as

(42) $${\bar{\sigma }}_{i}=\left[\sigma_{i,c,m}\right]\in R^{C\times M}.$$

The first absolute difference is defined as

(43) $$\Delta H_{i,c,t}=\left|H_{i,c,t}-H_{i,c,t-1}\right|,\;t=2,\ldots ,T,$$

with the first time step padded by repeating the first hidden feature, so $\Delta H_{i,c,1}=0$. Under the same segment weights, the weighted difference statistic is

(44) $$\delta_{i,c,m}=\frac{1}{q_{i,m}}\sum_{t=1}^{T}A_{i,m,t}\Delta H_{i,c,t}.$$

The standard-deviation branch and the difference branch may have different numerical scales. LPD-Net applies root-mean-square (RMS) scale matching to the difference statistic. For sample *i*,

(45) $$\mathrm{RMS}\left({\bar{\sigma }}_{i}\right)=\sqrt{\frac{1}{CM}\sum_{c=1}^{C}\sum_{m=1}^{M}\sigma_{i,c,m}^{2}},$$

(46) $$\mathrm{RMS}\left(\delta_{i}\right)=\sqrt{\frac{1}{CM}\sum_{c=1}^{C}\sum_{m=1}^{M}\delta_{i,c,m}^{2}}.$$

The scale-matched difference statistic is

(47) $${\tilde{\delta }}_{i}=\delta_{i}\cdot \frac{\mathrm{RMS}({\bar{\sigma }}_{i})}{\mathrm{RMS}(\delta_{i})+\epsilon }.$$

In implementation, the RMS scale factors are treated as detached normalization constants. The final segment-level representation is obtained by fusing the standard-deviation statistic and the scale-matched difference statistic:

(48) $$Z_{i}={\bar{\sigma }}_{i}+\alpha ⊙{\tilde{\delta }}_{i},\;Z_{i}\in R^{C\times M}.$$

Here, α is a channel-wise learnable gate and ⊙ denotes channel-wise multiplication. The gate is defined as

(49) $$\alpha =\alpha_{\max}\cdot \mathrm{sigmoid}\left(g\right),$$

where g is a learnable parameter, $\alpha_{\max}$ is the upper bound of the gate, and $\alpha \in R^{C}$. In this work, $\alpha_{\max}=1.0$, α is initialized to 0.05, and $\epsilon =10^{-6}$ is used in the normalization, variance, and RMS denominators. This gate acts on the feature-channel dimension, allowing the contribution of the difference statistic to be learned adaptively for each channel. During fusion, α is broadcast along the segment dimension. The resulting $Z\in R^{B\times C\times M}$ contains both fluctuation magnitude and local variation strength within dynamically aligned temporal-prototype representations.

### 3.3. Classification

After feature extraction, dynamic temporal alignment, and differential periodic representation, LPD-Net obtains the segment-level representation

(50) $$Z\in R^{B\times C\times M}.$$

For the *i*-th sample, the representation is flattened as

(51) $$z_{i}=\mathrm{vec}\left(Z_{i}\right)\in R^{CM}.$$

The classifier applies a linear mapping followed by softmax normalization:

(52) $$o_{i}=Wz_{i}+b,\;p_{i}=\mathrm{softmax}\left(o_{i}\right),$$

where $W\in R^{K\times CM}$, $b\in R^{K}$, and $p_{i}\in R^{K}$. The predicted class is

(53) $${\hat{y}}_{i}=\arg\max_{k}p_{i,k}.$$

The classifier receives a C×M-dimensional segment-level statistical representation for each sample. The *M* dimension of Z indexes dynamically aligned temporal-prototype-level representations, not fixed temporal intervals. Thus, the linear layer uses both feature-channel information and dynamic prototype-level information, and classification is based on the combination of statistical differences across channels and dynamically aligned prototypes.

### 3.4. Optimizer and Loss Function

LPD-Net uses cross-entropy as the classification objective. For a mini-batch, the classification loss is

(54) $$L_{\mathrm{cls}}=-\frac{1}{B}\sum_{i=1}^{B}\log p_{i,y_{i}},$$

where $p_{i,y_{i}}$ is the predicted probability of the true class for the *i*-th sample. This loss constrains the consistency between the model output and the ground-truth class label.

The temporal prototypes P are also constrained by a Soft-DTW alignment cost. This auxiliary alignment update is used only to regularize the learnable temporal prototypes and is separated from the classification loss update. Let sg(·) denote the stop-gradient operation. The prototype alignment cost is

(55) $$L_{\mathrm{align}}=\frac{1}{BT}\sum_{i=1}^{B}\mathrm{SoftDTW}\left(P^{T},\mathrm{sg}\left(H_{i}^{T}\right)\right).$$

Here, $P^{T}\in R^{M\times C}$ and $H_{i}^{T}\in R^{T\times C}$ are treated as two multivariate temporal sequences with feature dimension *C*. The factor 1/T normalizes the accumulated Soft-DTW cost with respect to the feature-sequence length. The stop-gradient operation prevents this auxiliary alignment loss from updating the backbone feature map, so the additional alignment update mainly stabilizes the temporal prototypes. Adam is used for optimization [26]. One iteration contains two gradient-update steps: first, $L_{\mathrm{cls}}$ updates all trainable parameters, including the backbone, the differential periodic representation module, the classification layer, and the temporal prototypes; second, under $\mathrm{sg}(H_{i}^{T})$, $L_{\mathrm{align}}$ applies an additional update to the temporal prototypes. In this way, class supervision and dynamic-alignment constraints jointly shape the feature representation of LPD-Net.

## 4. Experiments and Results

### 4.1. Experimental Setup and Evaluation Metrics

The experiments were implemented in Python v3.12.13 using PyTorch v2.6.0+cu126.

The experiments evaluate LPD-Net on IRPeriodic. The dataset contains eight geometry–micromotion object classes, with 640 samples per class and 5120 samples in total. Each input sample is an 80 s one-dimensional infrared radiant-intensity sequence sampled at 25 Hz, giving a sequence length of 2000 points. The task is formulated as eight-class classification on normalized univariate temporal sequences.

The dataset was divided into training, validation, and test subsets using a stratified trajectory-level protocol. Specifically, 56 AER trajectories were assigned to the training set, 12 trajectories to the validation set, and 12 trajectories to the test set. This resulted in 3584 training samples, 768 validation samples, and 768 test samples, corresponding to 448, 96, and 96 samples per class, respectively. The corresponding proportions are 70%, 15%, and 15%, as summarized in Table 5. Samples generated from the same AER trajectory were assigned to only one subset, so the same trajectory did not appear simultaneously in the training, validation, and test sets. This setting reduces trajectory leakage and evaluates classification performance under unseen AER trajectories.

All reported IRPeriodic results were averaged over five independent runs with different random seeds and are reported as mean ± standard deviation. Each sequence was independently normalized by min–max normalization before being fed into the classifiers. Because each sample is independently min–max normalized, the classification experiments evaluate normalized temporal morphology rather than absolute radiometric magnitude. The same normalization procedure was applied to LPD-Net and all baseline methods. The compared methods were trained and tested on the same data splits and with the same input normalization protocol. For neural-network models, the checkpoint with the best validation performance was used for test-set evaluation. MiniROCKET and MultiROCKET were evaluated using their random convolutional-kernel transform features followed by logistic-regression classifiers. The detailed hyperparameter configurations of LPD-Net and all benchmark methods are reported in Appendix A. Inference time denotes the average single-sample prediction time measured on the same hardware, excluding data loading; the unit is milliseconds. Classification metrics and parameter counts are reported to four decimal places, whereas inference time is reported to three decimal places in milliseconds to match the runtime measurement resolution and avoid over-reporting precision.

Performance is assessed using accuracy, macro-precision, macro-F1, and the Matthews correlation coefficient (MCC), which is included as a balanced classification metric [27]. For a multi-class confusion matrix C, class-wise precision and recall are

(56) $$P_{k}=\frac{C_{kk}}{\sum_{i}C_{ik}},\;R_{k}=\frac{C_{kk}}{\sum_{j}C_{kj}}.$$

The macro metrics are

(57) $$P_{\mathrm{macro}}=\frac{1}{K}\sum_{k=1}^{K}P_{k},\;F_{1,\mathrm{macro}}=\frac{1}{K}\sum_{k=1}^{K}\frac{2P_{k}R_{k}}{P_{k}+R_{k}}.$$

The multi-class MCC is

(58) $$\mathrm{MCC}=\frac{cs-\sum_{k}p_{k}t_{k}}{\sqrt{\left(s^{2}-\sum_{k}p_{k}^{2}\right)\left(s^{2}-\sum_{k}t_{k}^{2}\right)}}.$$

Here, *c* is the number of correctly classified samples, *s* is the total number of samples, $p_{k}$ is the number of samples predicted as class *k*, and $t_{k}$ is the number of true samples in class *k*. Unless otherwise specified, all results are reported as the mean ± standard deviation over five independent runs with different random seeds.

### 4.2. Overall Classification Performance on IRPeriodic

LPD-Net gives the best overall classification performance among the compared methods on IRPeriodic. As shown in Table 6, its accuracy reaches 0.8618 ± 0.0057, exceeding MultiROCKET by 3.10 percentage points and InceptionTime by 6.31 percentage points. The same tendency appears in macro-F1 and MCC. In particular, the MCC increases from 0.8069 ± 0.0044 for MultiROCKET to 0.8426 ± 0.0065 for LPD-Net, indicating that the improvement is not restricted to one favorable metric.

The baseline methods form three broad groups for time-series classification (TSC). MLP, FCN, and ResNet are included as standard deep-learning baselines [28]; LSTM is included as a representative recurrent baseline [29]; InceptionTime is included as a strong deep convolutional TSC baseline [30]; and ROCKET-type random convolutional-kernel transforms are included because of their strong TSC performance [31,32,33]. LSTM and FCN perform poorly, suggesting that a standard recurrent model or a relatively shallow fully convolutional architecture does not adequately capture the long-range modulation structure of the 2000-point sequences. MLP is better than these two methods, but still remains below the stronger convolutional and kernel-transform baselines. ROCKET-type transforms, ResNet, and InceptionTime are more competitive, which is consistent with their ability to extract multi-scale temporal patterns. However, these methods do not explicitly model sample-dependent temporal correspondence or segment-level periodic variation. These comparisons are consistent with the intended roles of the three modules: large kernels provide long temporal context, dynamic alignment reduces phase and segment-position mismatch, and differential periodic representation summarizes fluctuation magnitude and local variation strength.

This advantage should also be interpreted together with model size. Although LPD-Net has a substantially larger reported parameter count than all compared baselines, the within-architecture kernel-size ablation shows that increasing the parameter count alone does not guarantee higher accuracy. In particular, K199-99-9 has more parameters than K149-75-9 but achieves lower accuracy.

### 4.3. Class-Level Analysis

**Figure 7:**
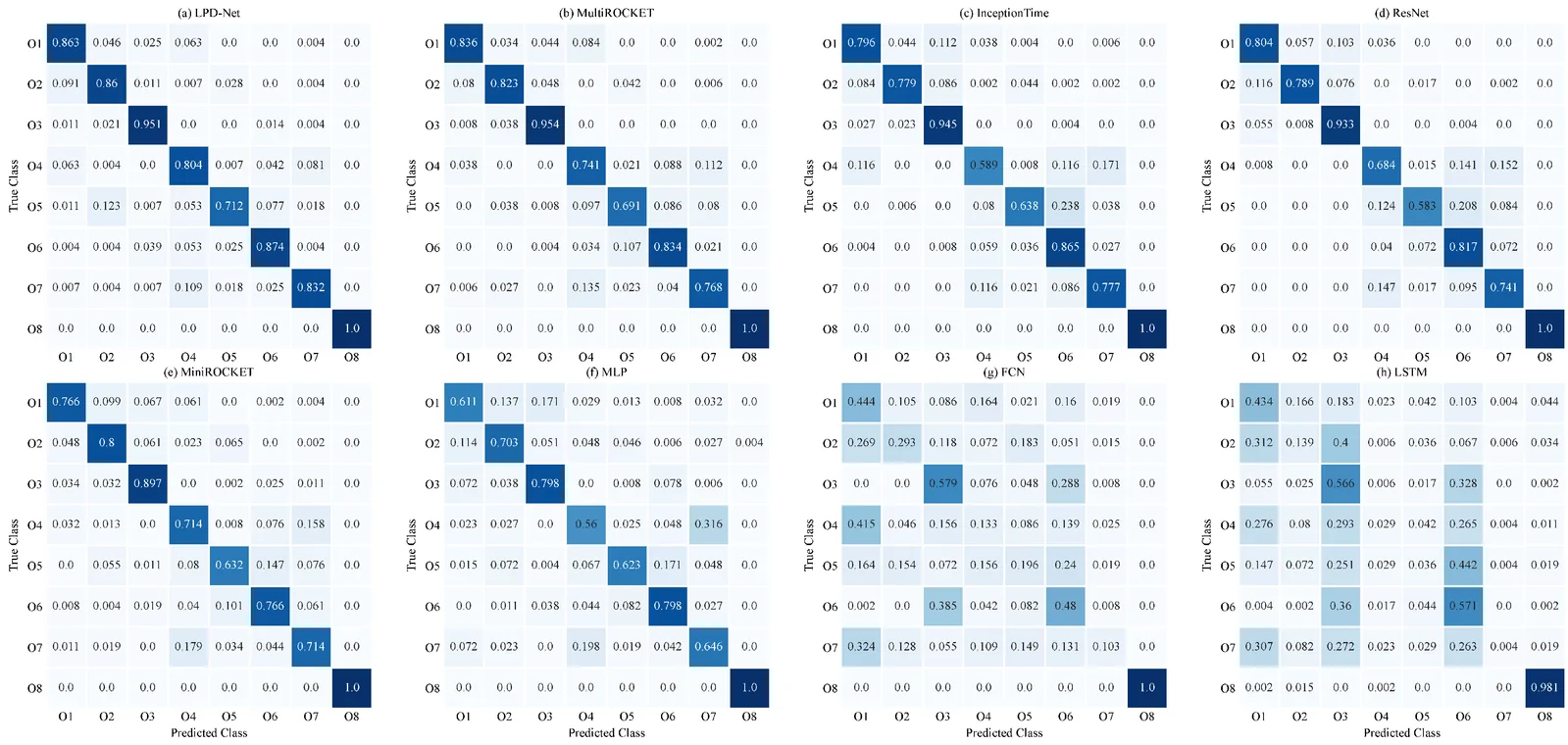
Row-normalized confusion matrices on IRPeriodic. Darker cells indicate higher class-conditional proportions.

Figure 7 shows that LPD-Net achieves clear diagonal dominance, with most residual errors concentrated in physically related class pairs, mainly $O_{5}$ with $O_{2}/O_{6}$ and $O_{4}$ with $O_{7}$. These confusions are consistent with overlapping geometry and micromotion-induced projected-area variations after normalization. The dominant confusions suggest that similar body geometry or micromotion can produce overlapping normalized temporal patterns under some viewing trajectories.

### 4.4. Sensitivity to Synthetic Additive White Gaussian Noise

To examine sensitivity to additive perturbations, additive white Gaussian noise (AWGN) is added to the simulated noise-free test sequences at SNR levels of 30 dB, 20 dB, and 10 dB. The SNR is defined using the average signal power of each sequence before per sequence min–max normalization. After noise injection, the perturbed sequences are normalized using the same independent min–max normalization protocol as the noise-free sequences. Unless otherwise stated, models are trained on the noise-free training set and evaluated on noise-contaminated test sets; this setting therefore evaluates sensitivity to synthetic additive noise without retraining. For a sequence $x={\left[x_{1},x_{2},\ldots ,x_{L}\right]}^{T}$, the noisy input is

(59) $$\tilde{x}=x+n,\;n_{t}∼N\left(0,\sigma_{n}^{2}\right),\;P_{x}=\frac{1}{L}\sum_{t=1}^{L}x_{t}^{2},\;\sigma_{n}^{2}=\frac{P_{x}}{10^{\mathrm{SNR}/10}}.$$

LPD-Net remains the best-performing method under all three noise levels. At 30 dB, its accuracy is 0.8276 ± 0.0057, higher than MiniROCKET by 7.13 percentage points and higher than InceptionTime by 10.71 percentage points. At 10 dB, LPD-Net still reaches 0.6884 ± 0.0176, whereas the best competing result is 0.5661 ± 0.0196 from InceptionTime.

The degradation pattern provides a similar indication. Methods that rely heavily on local waveform details or fixed transformations drop more rapidly as SNR decreases. The smaller degradation of LPD-Net may be associated with its segment-level aggregation: weighted standard deviation reduces dependence on individual points, while the difference branch captures local variation after dynamic alignment. This interpretation is consistent with the observed results, although the present experiment does not isolate the contribution of each mechanism under noise. The detailed AWGN sensitivity results are reported in Table 7.

### 4.5. Ablation Studies

The ablation studies separate the contributions of the three LPD-Net components: large-kernel residual feature extraction (L), prototype-guided dynamic temporal alignment (P), and differential periodic representation (D). For each ablated model, the corresponding component is replaced by a simpler counterpart while the other components and the training protocol are kept as close as possible to the full model.

#### 4.5.1. Contribution of L/P/D Modules

Removing the L module produces the largest drop, reducing accuracy from 0.8618 ± 0.0057 to 0.7377 ± 0.0167. This 12.41 percentage-point decrease shows that long-context feature extraction provides the main representational basis. Removing P reduces accuracy to 0.8444 ± 0.0106, indicating that dynamic alignment improves correspondence among latent micromotion stages. Removing D reduces accuracy to 0.8373 ± 0.0038, showing that standard-deviation statistics alone do not fully describe the local variation contained in the sequences. The corresponding L/P/D module ablation results are summarized in Table 8.

#### 4.5.2. Kernel Scale and Dynamic Segment Number

The kernel-size ablation further shows that performance is not a trivial consequence of using more parameters. K199-99-9 uses more parameters than K149-75-9 but obtains lower accuracy. Conversely, progressively shorter kernels reduce the effective temporal context and also reduce accuracy. The best setting therefore appears to be a balance between long-range context and local temporal resolution, as shown in Table 9.

The temporal-prototype ablation shows that both dynamic alignment and the number of learnable temporal prototypes matter. M=4 provides the best balance between under-partitioning and over-fragmentation. M=2 under-partitions the sequence, while M=8 and M=16 produce more fragmented prototype-level representations. Directly replacing prototype-guided temporal alignment with fixed equal-length segmentation also reduces accuracy. The temporal-prototype–number comparison is reported in Table 10, and the fixed-segmentation comparison is reported in Table 11.

Here, PTA denotes prototype-guided temporal alignment, and Std+Diff denotes the joint use of the standard-deviation and difference representations.

Compared with fixed segmentation, PTA produces sample-dependent alignment weights $A_{i}$.

#### 4.5.3. Differential Representation Variants

Table 12 evaluates the effects of fluctuation modeling, local-variation modeling, RMS-based scale matching, and adaptive channel-wise gating. Fixing the gate α degrades performance, while the raw adaptive Std+Diff variant yields a higher mean gate value but lower accuracy. This suggests that unnormalized local-variation features may introduce unstable channel-wise contributions. Therefore, the first-difference statistic should be scale-matched and adaptively gated before fusion.

Taken together, the ablation results indicate that the three modules contribute different aspects of the temporal representation. Long-context features provide the temporal basis, dynamic alignment improves correspondence among latent micromotion stages, and differential statistics enhance the representation of local periodic variation.

### 4.6. Auxiliary Evaluation on Public UCR Datasets

To examine whether the temporal representation is useful beyond the simulated infrared benchmark, LPD-Net is evaluated on 22 selected public UCR univariate time-series datasets. The UCR experiment is used as an auxiliary evaluation of the general univariate time-series representation. The UCR archive is a widely used benchmark collection for time-series classification [34], and recent large-scale benchmark studies have established UCR-based evaluation as a common protocol for TSC [35]. Recent time-series classification studies have also used related public benchmarks to evaluate general univariate sequence representations [36,37]. For each UCR dataset, the official training and test partitions were retained, and a stratified validation subset with a validation ratio of 0.2 was split from the official training set. As summarized in Table 13, LPD-Net achieves the highest mean accuracy of 0.9119 and the lowest mean classification error (MCE) of 0.0881. It also obtains 15 best results and 21 top-three placements. MCE is computed as

(60) $$\mathrm{MCE}=1-\frac{1}{N}\sum_{d=1}^{N}{\mathrm{Acc}}_{d}.$$

The UCR results should be interpreted at two levels. Recent large-scale evaluations show that modern convolutional-kernel and hybrid methods remain highly competitive for TSC [35]. At the aggregate level, LPD-Net improves mean accuracy over MultiROCKET by 1.19 percentage points and over InceptionTime by 1.20 percentage points. It also appears in the top three in 21 of the 22 datasets, indicating broad competitiveness across the selected datasets with different temporal lengths, class numbers, and waveform characteristics. At the dataset level, however, LPD-Net is not uniformly superior. On datasets such as ECG200, ECG5000, FreezerRegularTrain, and HandOutlines, other methods match or exceed its result. This pattern is normal in time-series classification and suggests that LPD-Net is broadly competitive rather than universally dominant.

The datasets on which LPD-Net performs well provide useful support for its design. Strong results on FordA, FordB, Herring, and several MiddlePhalanx datasets indicate that the model is effective when class differences are expressed through waveform morphology, local deformation, and phase-dependent temporal structure. These properties are close to the intended role of large-kernel extraction and dynamic temporal alignment. The UCR results therefore provide auxiliary evidence that the proposed representation is not narrowly tuned to IRPeriodic-like sequences on the selected public datasets. The full dataset-level UCR results are provided in Appendix A.

## 5. Discussion

The results are consistent with the physical motivation of LPD-Net. Infrared radiant-intensity sequences contain long-duration modulation associated with object attitude, periodic fluctuation caused by micromotion, local changes from projected area and transient temperature evolution, and attenuation changes introduced by ground-based observation geometry. LPD-Net reflects these properties through three operations: large-kernel convolution captures long-context waveform information, prototype-guided alignment reduces temporal-stage mismatch across samples, and the differential periodic representation summarizes fluctuation magnitude and adjacent-time variation within aligned segments. Improvements in accuracy, macro-F1, and MCC, together with the confusion-matrix, AWGN, and ablation results, suggest that these modules provide complementary forms of temporal representation rather than a gain from a single component.

IRPeriodic provides a controlled physics-informed benchmark for evaluating long-duration temporal classification under variations in object geometry, micromotion, thermal state, ground-based viewing geometry, and atmospheric transmission. The trajectory-level split further evaluates generalization to unseen prescribed AER trajectories, while the UCR experiments provide complementary evidence on the general temporal representation. Future work will extend the present framework toward specific ground-observation sites and instruments by incorporating site-specific observing conditions and instrument parameters, and will further evaluate the method using measured or semi-measured infrared data and more complex target and visibility conditions.

## 6. Conclusions

This study developed a physics-informed simulation and time-series classification framework for ground-based infrared radiant-intensity sequences of space objects. The sequence-generation model couples AER-driven observation geometry, micromotion attitude, projected-area variation, transient thermal radiation, and MODTRAN-derived atmospheric attenuation to construct IRPeriodic, an eight-class simulated benchmark of long-duration univariate infrared sequences. For classification, LPD-Net combines large-kernel residual feature extraction, prototype-guided dynamic temporal alignment, and differential periodic representation to model long-range waveform morphology, sample-dependent temporal correspondence, and segment-level local variation.

On IRPeriodic, LPD-Net outperformed the evaluated neural-network and ROCKET-type baselines in accuracy, macro-F1, and MCC. Ablation and synthetic-noise sensitivity analyses indicated that long-context feature extraction provides the main performance gain, while dynamic alignment and differential periodic statistics add complementary improvements. These results demonstrate that matching the temporal representation to the physically induced long-range modulation, phase-dependent shifts, and local variations is effective for the considered ground-based infrared radiant-intensity classification problem. The resulting physics-informed simulation and classification framework provides a methodological basis for subsequent evaluation using measured infrared observations under site- and instrument-specific observation conditions. 

## Figures and Tables

**Table 1:** Simulation parameters of the eight object classes.

Parameter	$O_{1}$	$O_{2}$	$O_{3}$	$O_{4}$	$O_{5}$	$O_{6}$	$O_{7}$	$O_{8}$
Geometry	Flat-base cone	Cone–cyl. composite	Spherical base cone	Flat-base cone	Cone–cyl. composite	Spherical base cone	Cylinder	Hexagonal prism
Micromotion mode	Precession	Precession	Precession	Tumbling	Tumbling	Tumbling	Tumbling	Tumbling
Height *H*/m	1∼2	–	–	1∼2	–	–	1∼2	1∼2
Cone height $H_{\mathrm{cone}}$/m	–	1∼2	1∼2	–	1∼2	1∼2	–	–
Cylinder height $H_{\mathrm{cyl}}$/m	–	1∼2	–	–	1∼2	–	–	–
Radius *r*/m	0.25∼0.50	0.25∼0.50	–	0.25∼0.50	0.25∼0.50	–	0.25∼0.50	–
Spherical radius $r_{\mathrm{sph}}$/m	–	–	0.25∼0.50	–	–	0.25∼0.50	–	–
Hexagonal radius $r_{\mathrm{hex}}$/m	–	–	–	–	–	–	–	0.25∼0.50
Precession angle $\theta_{p}$/rad	π/60, π/18 π/10, π/6	π/60, π/18 π/10, π/6	π/60, π/18 π/10, π/6	–	–	–	–	–
Tumbling-axis inclination $\theta_{t}$/rad	–	–	–	π/60, π/18 π/10, π/6	π/60, π/18 π/10, π/6	π/60, π/18 π/10, π/6	π/60, π/18 π/10, π/6	π/60, π/18 π/10, π/6
Precession angular velocity $\omega_{p}$/rad $s^{-1}$	0.2π, 0.5π	0.2π, 0.5π	0.2π, 0.5π	–	–	–	–	–
Spin angular velocity $\omega_{s}$/rad $s^{-1}$	4π	4π	4π	–	–	–	–	–
Tumbling angular velocity $\omega_{t}$/rad $s^{-1}$	–	–	–	0.2π 0.5π	0.2π 0.5π	0.2π 0.5π	0.2π 0.5π	0.2π 0.5π
Coating material	Gray ${\mathrm{TiO}}_{2}$ paint	Gray ${\mathrm{TiO}}_{2}$ paint	Gray ${\mathrm{TiO}}_{2}$ paint	Aluminum	Al foil/ Al	Polished metal	Black paint	Al coating/ Al paint
Thickness/mm	1	1	1	1	1	1	1	1
Emissivity ε	0.87	0.87	0.87	0.036	0.036	0.028	0.874	0.45
Solar absorptivity $\alpha_{s}$	0.87	0.87	0.87	0.192	0.192	0.301	0.975	0.54
Density/kg $m^{-3}$	4260	4260	4260	2710	2700	7800	1300	2700
Specific heat/J ${\mathrm{kg}}^{-1}K^{-1}$	811	811	811	880	900	500	910	900

**Table 2:** Dataset parameter settings.

Parameter	Value	Description
Spectral band	8–12 μm	Band for directional radiant-intensity calculation
Atmospheric setting	MODTRAN baseline setting	Mid-latitude winter atmosphere, rural aerosol, 20 km visibility, and 3.2 km observer altitude
Sequence setting	25 Hz, 80 s, L=2000, K=8	Equal-length infrared radiant-intensity sequence
Dataset size	640 samples per class, 5120 total	Generated from angle and angular-velocity combinations across 80 prescribed AER trajectories

**Table 3:** Structural parameters of the large-kernel residual convolution block.

Layer	Kernel Length	Stride	Output Channels	Norm.	Activation
Conv1	149	1	$C_{b}$	BN	ReLU
Conv2	75	1	$C_{b}$	BN	ReLU
Conv3	9	1	$C_{b}$	BN	–
Skip	1	1	$C_{b}$	BN	–

**Table 4:** Backbone channel configuration of LPD-Net.

Residual Block	Output Channels
Block 1	64
Block 2	128
Block 3	128
Block 4	128
Block 5	128
Block 6	128

**Table 5:** Dataset split used for IRPeriodic experiments.

Split	Traj.	Samples/Class	Total Samples	Proportion
Training	56	448	3584	70%
Validation	12	96	768	15%
Test	12	96	768	15%
Total	80	640	5120	100%

**Table 6:** Overall classification results on IRPeriodic. Values are reported as mean ± standard deviation.

Method	Acc	$P_{\mathrm{macro}}$	$F_{1,\mathrm{macro}}$	MCC	Params (M)	Inference Time (ms)
**LPD-Net**	**0.8618** ± **0.0057**	**0.8650** ± **0.0057**	**0.8615** ± **0.0061**	**0.8426** ± **0.0065**	18.3072	1.247 ± 0.023
MultiROCKET	0.8308 ± 0.0039	0.8318 ± 0.0035	0.8305 ± 0.0038	0.8069 ± 0.0044	0.6398	9.450 ± 0.090
InceptionTime	0.7987 ± 0.0136	0.8069 ± 0.0141	0.7967 ± 0.0146	0.7717 ± 0.0153	0.4212	0.135 ± 0.002
ResNet	0.7939 ± 0.0174	0.8041 ± 0.0183	0.7934 ± 0.0179	0.7661 ± 0.0199	1.4355	0.507 ± 0.029
MiniROCKET	0.7861 ± 0.0077	0.7885 ± 0.0077	0.7859 ± 0.0078	0.7559 ± 0.0088	**0.0800**	1.720 ± 0.520
MLP	0.7174 ± 0.0181	0.7193 ± 0.0184	0.7162 ± 0.0186	0.6776 ± 0.0206	1.5055	**0.015** ± **0.003**
FCN	0.4034 ± 0.0030	0.4200 ± 0.0129	0.3847 ± 0.0047	0.3233 ± 0.0028	0.2659	0.072 ± 0.004
LSTM	0.3450 ± 0.0092	0.2899 ± 0.0351	0.2844 ± 0.0132	0.2652 ± 0.0107	0.2002	0.121 ± 0.001

Note: Bold values indicate the best result in each column.

**Table 7:** Accuracy under AWGN perturbation at different SNR levels.

Method	30 dB	20 dB	10 dB
MLP	0.6608 ± 0.0188	0.6353 ± 0.0112	0.4839 ± 0.0177
LSTM	0.3445 ± 0.0088	0.3376 ± 0.0161	0.3153 ± 0.0147
FCN	0.3763 ± 0.0105	0.3587 ± 0.0134	0.3418 ± 0.0119
ResNet	0.6608 ± 0.0305	0.5739 ± 0.0262	0.4434 ± 0.0126
InceptionTime	0.7205 ± 0.0313	0.6684 ± 0.0239	0.5661 ± 0.0196
MiniROCKET	0.7563 ± 0.0072	0.6608 ± 0.0058	0.5416 ± 0.0064
MultiROCKET	0.6800 ± 0.0107	0.5916 ± 0.0052	0.5011 ± 0.0063
**LPD-Net**	**0.8276 ± 0.0057**	**0.7721 ± 0.0107**	**0.6884 ± 0.0176**

Note: Bold values indicate the best result in each column.

**Table 8:** Core module ablation of LPD-Net.

Configuration	L	P	D	Acc	$P_{\mathrm{macro}}$	$F_{1,\mathrm{macro}}$	MCC	Params (M)	Inference Time (ms)
**LPD-Net**	✓	✓	✓	**0.8618± 0.0057**	**0.8650 ± 0.0057**	**0.8615 ± 0.0061**	**0.8426 ± 0.0065**	18.3072	1.247 ± 0.023
LPD-Net w/o P	✓	-	✓	0.8444 ± 0.0106	0.8464 ± 0.0101	0.8438 ± 0.0105	0.8240 ± 0.0121	18.3072	1.062 ± 0.002
LPD-Net w/o D	✓	✓	-	0.8373 ± 0.0038	0.8398 ± 0.0044	0.8368 ± 0.0040	0.8145 ± 0.0044	18.3071	1.187 ± 0.001
LPD-Net w/o L	-	✓	✓	0.7377 ± 0.0167	0.7412 ± 0.0144	0.7368 ± 0.0166	0.7010 ± 0.0188	**0.5499**	**0.343 ± 0.049**

Note: ✓ indicates that the corresponding module is included, whereas – indicates that it is removed. Bold values indicate the best result in each column.

**Table 9:** Large-kernel configuration ablation.

Kernel Setting	Acc	$P_{\mathrm{macro}}$	$F_{1,\mathrm{macro}}$	MCC	Params (M)	Inference Time (ms)
K199-99-9	0.8390 ± 0.0112	0.8412 ± 0.0111	0.8384 ± 0.0110	0.8165 ± 0.0128	24.0613	1.545 ± 0.028
**K149-75-9**	**0.8618 ± 0.0057**	**0.8650 ± 0.0057**	**0.8615 ± 0.0061**	**0.8426± 0.0065**	18.3072	1.247 ± 0.023
K99-49-9	0.8355 ± 0.0122	0.8388 ± 0.0125	0.8347 ± 0.0119	0.8127 ± 0.0141	12.3812	0.985 ± 0.002
K49-25-5	0.8389 ± 0.0078	0.8424 ± 0.0067	0.8386 ± 0.0079	0.8165 ± 0.0087	6.2832	0.701 ± 0.005
K25-13-5	0.8200 ± 0.0129	0.8239 ± 0.0121	0.8193 ± 0.0132	0.7950 ± 0.0145	3.4800	0.577 ± 0.001
K19-9-5	0.8147 ± 0.0093	0.8184 ± 0.0098	0.8143 ± 0.0094	0.7889 ± 0.0106	2.6931	0.535 ± 0.004
K9-7-3	0.7811 ± 0.0167	0.7863 ± 0.0162	0.7815 ± 0.0172	0.7504 ± 0.0190	**1.6112**	**0.487 ± 0.005**

Note: Bold values indicate the best result in each column.

**Table 10:** Ablation of the number of temporal prototypes *M*.

Configuration	Acc	$P_{\mathrm{macro}}$	$F_{1,\mathrm{macro}}$	MCC	Params (M)	Inference Time (ms)
M = 2	0.8439 ± 0.0114	0.8478 ± 0.0106	0.8438 ± 0.0110	0.8236 ± 0.0130	**18.3049**	**1.189 ± 0.005**
**M = 4**	**0.8618 ± 0.0057**	**0.8650 ± 0.0057**	**0.8615 ± 0.0061**	**0.8426 ± 0.0065**	18.3072	1.247 ± 0.023
M = 8	0.8491 ± 0.0084	0.8512 ± 0.0089	0.8485 ± 0.0082	0.8280 ± 0.0096	18.3118	1.376 ± 0.004
M = 16	0.8250 ± 0.0092	0.8278 ± 0.0081	0.8245 ± 0.0087	0.8005 ± 0.0105	18.3210	1.641 ± 0.005

Note: Bold values indicate the best result in each column.

**Table 11:** Ablation of dynamic alignment versus fixed segmentation.

Configuration	Acc	$P_{\mathrm{macro}}$	$F_{1,\mathrm{macro}}$	MCC	Params (M)	Inference Time (ms)
Fixed-Std+Diff	0.8444 ± 0.0106	0.8464 ± 0.0101	0.8438 ± 0.0105	0.8240 ± 0.0121	**18.3072**	**1.062 ± 0.002**
**PTA-Std+Diff**	**0.8618 ± 0.0057**	**0.8650 ± 0.0057**	**0.8615 ± 0.0061**	**0.8426 ± 0.0065**	**18.3072**	1.247 ± 0.023

Note: Bold values indicate the best result in each column.

**Table 12:** Internal ablation of the differential periodic representation module.

Configuration	Acc	$P_{\mathrm{macro}}$	$F_{1,\mathrm{macro}}$	MCC	Gate Mean	Params (M)	Inference Time (ms)
Std baseline	0.8373 ± 0.0038	0.8398 ± 0.0044	0.8368 ± 0.0040	0.8145 ± 0.0044	-	**18.3071**	**1.1875 ± 0.001**
Scaled-Adaptive	0.8483 ± 0.0055	0.8520 ± 0.0067	0.8479 ± 0.0061	0.8287 ± 0.0063	0.0808 ± 0.0046	18.3072	1.208 ± 0.022
Std+Max							
Raw-Adaptive	0.8431 ± 0.0053	0.8451 ± 0.0060	0.8428 ± 0.0055	0.8224 ± 0.0061	0.0924 ± 0.0116	18.3072	1.257 ± 0.042
Std+Diff							
Scaled-Fixed	0.8505 ± 0.0103	0.8428 ± 0.0098	0.8402 ± 0.0102	0.8310 ± 0.0117	0.0500 ± 0.0000	**18.3071**	1.280 ± 0.001
Std+Diff							
**Scaled-Adaptive**	**0.8618 ± 0.0057**	**0.8650 ± 0.0057**	**0.86156 ± 0.00616**	**0.8426 ± 0.006**	0.0822 ± 0.0050	18.3072	1.247 ± 0.023
**Std+Diff**							

Note: Bold values indicate the best result in each column.

**Table 13:** Summary of public UCR dataset results.

Method	Mean Acc	MCE	Best Count	Top-3 Count
MLP	0.8412	0.1588	2	5
LSTM	0.8383	0.1617	2	2
FCN	0.8918	0.1082	8	9
ResNet	0.8803	0.1197	5	6
InceptionTime	0.8999	0.1001	9	12
MiniROCKET	0.8925	0.1075	7	12
MultiROCKET	0.9000	0.1000	9	17
**LPD-Net**	**0.9119**	**0.0881**	**15**	**21**

Note: Bold values indicate the best result in each column.

## Data Availability

The internally generated IRPeriodic dataset and implementation code are available from the corresponding author upon reasonable request, subject to institutional and project-related restrictions. The public UCR datasets used in this study are available from the UCR Time Series Classification Archive.

## Appendix A. Hyperparameter Configurations and Full UCR Dataset-Level Accuracy Results

Table A1 lists the hyperparameter configurations used for LPD-Net and the benchmark methods.

**Table A1:** Hyperparameter configurations of benchmark methods.

Method	Hyperparameter Configurations
MLP	One hidden layer with 500 neurons; dropout = 0.2; Adam optimizer, learning rate = $1\times 10^{-4}$, weight decay = $1\times 10^{-4}$, batch size = 64, epochs = 500
LSTM	Hidden size = 128, number of layers = 2; dropout = 0.2; Adam optimizer, learning rate = $1\times 10^{-4}$, weight decay = $1\times 10^{-4}$, batch size = 64, epochs = 500
FCN	Three convolutional layers with kernel sizes 9-5-3; Adam optimizer, learning rate = $1\times 10^{-4}$, weight decay = $1\times 10^{-4}$, batch size = 64, epochs = 500
ResNet	Six-layer temporal ResNet with convolutional kernel sizes 9-5-3; Adam optimizer, learning rate = $1\times 10^{-4}$, weight decay = $1\times 10^{-4}$, batch size = 64, epochs = 500
InceptionTime	Depth = 6, output channels = 32, bottleneck channels = 32; dropout = 0.2; Adam optimizer, learning rate = $1\times 10^{-4}$, weight decay = $1\times 10^{-4}$, batch size = 64, epochs = 500
MiniROCKET	MiniROCKET transform with 10,000 kernels and max dilations per kernel = 32; StandardScaler without centering; logistic regression classifier, *C* = 1.0, solver = lbfgs, max iterations = 2000
MultiROCKET	MultiROCKET transform with 6250 kernels and max dilations per kernel = 32; StandardScaler without centering; logistic regression classifier, *C* = 1.0, solver = lbfgs, max iterations = 2000
LPD-Net	Large-kernel residual backbone with kernel sizes 149-75-9; 4 learnable temporal prototypes and prototype-guided Soft-DTW-based soft alignment; differential periodic representation with hidden-feature absolute first difference, first-step padding by repeating the first hidden feature, RMS scale matching, channel-wise learnable residual gate with $\alpha_{\max}=1.0$ and gate initialized to 0.05, $\epsilon =10^{-6}$; Adam optimizer, learning rate = $1\times 10^{-4}$, weight decay = $1\times 10^{-4}$, batch size = 64, epochs = 500

Table A2 reports the full dataset-level UCR accuracy results and aggregate statistics.

**Table A2:** Dataset-level UCR accuracy results and aggregate statistics. Dataset rows report accuracy; ties are retained, and Top-3 Count uses competition ranking.

Dataset/Statistic	MLP	LSTM	FCN	ResNet	Inception Time	Mini ROCKET	Multi ROCKET	LPD-Net
Coffee	**1.0000**	**1.0000**	**1.0000**	**1.0000**	**1.0000**	**1.0000**	**1.0000**	**1.0000**
Earthquakes	0.7920	0.6763	**0.8010**	0.7482	0.7482	0.7410	0.7482	0.7860
ECG200	0.9200	0.9000	0.9000	0.8740	**0.9300**	0.9100	0.9200	0.8900
ECG5000	0.9362	0.9320	0.9410	0.9358	0.9422	0.9451	**0.9464**	0.9440
FordA	0.8394	0.7894	0.9165	0.9439	0.9583	0.9508	0.9576	**0.9614**
FordB	0.7136	0.7062	0.8830	0.8420	0.8605	0.8198	0.8383	**0.9000**
FreezerRegularTrain	0.8589	0.9319	0.9966	0.9940	0.9968	**0.9996**	**0.9996**	0.9989
GunPoint	0.9330	0.9467	**1.0000**	0.9933	**1.0000**	0.9933	**1.0000**	**1.0000**
GunPointAgeSpan	0.8829	0.9430	0.9918	0.9968	0.9905	0.9968	**1.0000**	**1.0000**
GunPointMaleVersusFemale	0.9304	0.9968	0.9962	0.9937	**1.0000**	**1.0000**	**1.0000**	**1.0000**
GunPointOldVersusYoung	0.7873	**1.0000**	0.9968	**1.0000**	**1.0000**	**1.0000**	**1.0000**	**1.0000**
Ham	0.7140	0.6667	0.7620	**0.8190**	0.7714	0.7238	0.7429	**0.8190**
HandOutlines	0.9135	0.9189	0.8114	0.9162	**0.9595**	0.9378	0.9514	0.9432
Herring	0.6870	0.6406	0.7030	0.6000	0.7031	0.7188	0.7344	**0.7656**
ItalyPowerDemand	0.9660	0.9553	**0.9700**	0.9650	0.9660	0.9631	0.9679	0.9699
MiddlePhalanxOutlineAgeGroup	0.6169	0.5260	0.5584	0.5909	0.5779	0.5584	0.6169	**0.6364**
MiddlePhalanxOutlineCorrect	0.7835	0.7629	0.8110	0.8261	0.8351	0.8488	0.8591	**0.8660**
MiddlePhalanxTW	**0.6104**	0.5325	**0.6104**	0.5000	0.5779	0.5455	0.5390	0.5974
Plane	0.9810	0.9809	**1.0000**	**1.0000**	**1.0000**	**1.0000**	**1.0000**	**1.0000**
Strawberry	0.9670	0.9622	0.9697	0.9800	**0.9838**	**0.9838**	0.9811	**0.9838**
Trace	0.8200	0.7700	**1.0000**	**1.0000**	**1.0000**	**1.0000**	**1.0000**	**1.0000**
TwoLeadECG	0.8530	0.9043	**1.0000**	0.8481	0.9956	0.9982	0.9982	**1.0000**
Best Count	2	2	8	5	9	7	9	**15**
Mean Acc	0.8412	0.8383	0.8918	0.8803	0.8999	0.8925	0.9000	**0.9119**
MCE	0.1588	0.1617	0.1082	0.1197	0.1001	0.1075	0.1000	**0.0881**
Top-3 Count	5	2	9	6	12	12	17	**21**

Note: Bold values indicate the best result(s) for each dataset or aggregate statistic; ties are retained.
